# *TNF-alpha* Promoter Single-Nucleotide Polymorphisms and Inflammatory Bowel Diseases in Romania: Association with Disease Susceptibility and Clinical Features

**DOI:** 10.3390/jcm15031042

**Published:** 2026-01-28

**Authors:** Cristian George Țieranu, Luis Ovidiu Popa, Ioana Țieranu, Monica Irina Duțescu, Carmen Monica Preda, Andrei Ovidiu Olteanu, Cristian Valentin Toma, Adrian Săftoiu, Olivia Mihaela Popa

**Affiliations:** 1 Department of Gastroenterology, “Carol Davila” University of Medicine and Pharmacy, 050474 Bucharest, Romania; cristian.tieranu@umfcd.ro (C.G.Ț.); carmen.preda@umfcd.ro (C.M.P.); adrian.saftoiu@umfcd.ro (A.S.); 2 Department of Gastroenterology, “Elias” Emergency University Hospital, 011461 Bucharest, Romania; 3 Molecular Biology Department, “Grigore Antipa” National Museum of Natural History, 011341 Bucharest, Romania; popaluis@antipa.ro; 4 Department of Pediatrics, “Marie Sklodowska Curie” Clinical Emergency Hospital for Children, 041434 Bucharest, Romania; dr.cindeaioana@yahoo.ro; 5 National HLA Laboratory, ‘‘Prof. Dr. Constantin T. Nicolau’’ National Institute of Blood Transfusion, 011155 Bucharest, Romania; monica_dutescu@yahoo.com; 6 Department of Gastroenterology, Fundeni Clinical Institute, 022328 Bucharest, Romania; 7 Department of Urology, “Carol Davila” University of Medicine and Pharmacy, 050474 Bucharest, Romania; 8 Department of Pathophysiology and Immunology, “Carol Davila” University of Medicine and Pharmacy, 050474 Bucharest, Romania; olivia.popa@umfcd.ro

**Keywords:** inflammatory bowel disease, ulcerative colitis, Crohn’s disease, TNF-alpha, single-nucleotide polymorphism, extraintestinal manifestations

## Abstract

**Background/Objectives**: Tumor necrosis factor alpha (TNF-alpha) plays a key role in systemic inflammation in multiple disorders, including inflammatory bowel diseases (IBDs). Our purpose was to investigate the contribution of two promoter single-nucleotide polymorphisms (rs361525/–238G/A and rs1800629/–308G/A) to disease susceptibility, clinical features, and response to biologic therapy in a cohort of Romanian patients with IBDs. **Methods**: A total of 198 patients with IBDs, 106 with Crohn’s disease (CD) and 92 with ulcerative colitis (UC), as well as 160 healthy controls, all Caucasians of Romanian origin, were genotyped using TaqMan Allelic Discrimination Assays. Phenotypical and anti-TNF treatment characteristics of the patients with IBDs were recorded. Statistical analyses were performed using OpenEpi and PLINK v1.07 software. **Results**: We found a significantly higher frequency of the minor allele A of rs361525 in patients with CD than in the controls (6.6% vs. 2.2%, *p* = 0.01, OR = 3.16). Half of the patients with extraintestinal manifestations (EIMs) had at least one copy of the rs1800629 A allele compared with approximately 10% of patients without EIM (*p* = 1 × 10^−4^, OR 9.58 for UC and *p* = 9 × 10^−4^, OR 6.60 for CD). In the whole IBD group of patients, the carriers of the minor allele (AA+GA) for both SNPs studied (rs1800629 and rs361525) were significantly more likely to have EIM associated with IBDs (*p* = 3 × 10^−7^, OR 7.87; *p* = 0.03, OR 3.02, respectively). In patients with UC, the analysis according to disease extension revealed that the frequency of the minor allele of rs1800629 was significantly higher in the subgroup with the E2 phenotype compared to the E1 and E3 phenotypes (16.6% versus 5.6%, *p* = 0.02, OR 3.32). **Conclusions**: These findings highlight the role of genetic *TNF-alpha* variants in disease susceptibility, phenotype, and systemic involvement, supporting their potential relevance in understanding IBD heterogeneity.

## 1. Introduction

Inflammatory bowel diseases (IBDs) are chronic inflammatory gastrointestinal (GI) diseases with a polygenic architecture and are strongly influenced by environmental factors [1]. They are mainly represented by ulcerative colitis (UC) and Crohn’s disease (CD), predominantly affecting the young population, thus generating a public health issue [2]. Recent hypotheses regarding their etiology imply exposure to environmental factors and dysbiosis as determinants of abnormal immune response in a genetically predisposed host [3]. Patients carrying alleles predisposing a person to IBDs have a disproportionate immune response to intestinal microbiota, thus developing IBD symptoms [4]. Linkage and genome-wide studies have repeatedly implicated the MHC region on 6p21 (IBD3), which harbors the TNF locus, as a contributor to IBD susceptibility, alongside non-MHC loci such as NOD2 (IBD1) [5].

Tumor necrosis factor alpha (TNF-alpha) plays a key role in systemic inflammation in multiple disorders such as Behcet’s disease, rheumatoid arthritis, nephrotic syndrome, and IBDs [6,7,8]. Identification of TNF-alpha as an important member of the cytokine family responsible for the inflammation seen in IBDs has led to the development of anti-TNF-alpha molecules, which, until recently, have been the most potent agents available for the treatment of these disorders. Regardless of these therapeutic advances, a significant proportion of patients fail to respond without any identified cause [9]. This observation has led to concurrent research, including genetic variations analysis.

The *TNF-alpha* gene is located on chromosome 6 in the p21.3 region, between the HLA-B and HLA-DR loci. The most studied polymorphisms are located in the promoter region of the gene [10]. Genetic variations in the *TNF-alpha* gene at positions -308 and -238 result in two allelic forms, in which the presence of guanine (G) defines the common variant, and the presence of adenine (A) defines the less common one. Promoter single-nucleotide polymorphisms (SNPs) at −308 (rs1800629, G>A) and −238 (rs361525, G>A) have been studied because of in vitro evidence of altered transcription and because they point towards common haplotypes within the dense TNF block [11]. At least for the –308A allele, increased gene transcription has been reported compared to the common variant, resulting in 6- to 7-fold higher levels of TNF-alpha production [12,13].

Even though genetic associations have been reported between promoter region polymorphisms of the *TNF-alpha* gene and IBDs, they are discrepant and inconsistently replicated [14,15,16,17]. Differences in results have been attributed to genetic ancestral variations in these populations [18]. Interestingly, there are notable differences in terms of epidemiology and disease phenotype between Western European and Asian patients with IBDs, supporting genetics as a key determinant of IBD etiopathogenesis [19,20].

Data from Eastern Europe remain comparatively sparse despite the rising IBD incidence and potentially distinct linkage disequilibrium (LD) patterns across the extended MHC, underscoring the need for ancestry-matched estimates [21].

As part of our group’s ongoing work on IBD genetics in Romania, we previously reported that *IL4* gene polymorphisms are associated with IBD risk and selected clinical features in Romanian patients, supporting further evaluation of cytokine-pathway variants in this population [22].

Given the potential implications of *TNF-alpha* gene polymorphisms in the pathogenesis and response to treatment of IBDs, we aimed to investigate the contribution of rs361525 and rs1800629 polymorphisms in disease susceptibility, clinical features, and response to anti-TNF therapy in a cohort of Romanian patients with IBDs.

## 2. Materials and Methods

### 2.1. Study Population

Patients with IBD were consecutively recruited in two referral centers: “Elias” Emergency University Hospital (2017–2024) and Fundeni Clinical Institute (2019–2022), both located in Bucharest, Romania. Diagnoses of IBDs, CD, and UC were established based on guidelines [23,24].

A total of 198 patients with IBDs, unrelated and of Romanian ancestry, were included in the study: 106 were patients with CD (57 men, mean age 29.7 years), and 92 were patients with UC (55 men, mean age 34.4 years). The Romanian ethnicity was certified based on the surname of the subjects, accompanied by a self-reported statement that at least three ancestral generations had lived within Romanian territory.

The inclusion criteria were age above 18 years, certified Romanian heritage, and an established diagnosis of IBDs based on the above-mentioned recommendations. Although this approach reduces the likelihood of population stratification, residual substructure could not be completely excluded.

Exclusion criteria were refusal to sign the informed consent and other inflammatory ileitis/colitis not confirmed to be IBDs.

The control group consisted of 160 healthy, unrelated, asymptomatic adult subjects, serving as blood donors (104 men), recruited from the “Prof. Dr. C. T. Nicolau” National Transfusion Hematology Institute of Bucharest. Controls were recruited from the same geographic area. Age at recruitment for controls was not systematically recorded because germline genotype frequencies are not expected to vary with age.

### 2.2. Data Collection

The Montreal classification was used for both UC and CD [25]. The following clinical features were recorded: sex, age, age at diagnosis, disease location for CD (ileal, ileocolonic, colonic, and upper GI tract), disease behavior for CD (inflammatory, stricturing, penetrating), extent of UC (proctitis, left-side colitis, pancolitis), extraintestinal manifestations (presence or absence), need for colectomy in UC. The extraintestinal manifestations (EIMs) noted were ocular (uveitis), cutaneous (erythema nodosum and pyoderma gangrenosum), arthritis, either axial or peripheral. The standard investigations used in these patients were upper gastrointestinal endoscopy, ileocolonoscopy, magnetic resonance enterography, biopsy sampling, and histopathological examinations to support the diagnosis of IBDs.

For the purpose of this study, information regarding anti-TNF agents was collected: response to anti-TNF treatment (primary non-responder vs. responder, as determined by the treating physician) and adverse events during treatment, defined as infusion-related reactions and infections associated with treatment contributing to treatment cessation. This information was obtained by reviewing the patients’ medical records.

### 2.3. DNA Extraction and Genotyping

Genomic DNA was obtained from blood samples extracted on EDTA-treated tubes using the QIAamp DNA Blood Mini Kit (Qiagen, Hilden, Germany) according to the manufacturer’s protocol [26].

Two single-nucleotide polymorphisms of the TNF-alpha gene were selected for this study: rs361525 and rs1800629. The SNP genotyping was performed by real-time polymerase chain reaction using TaqMan Allelic Discrimination Assays C_2215707_10 and C_7514879_10 on a 7300 series Real-Time PCR System (Applied Biosystems by Thermo Fisher Scientific, Foster City, CA, USA) following the manufacturer’s instructions.

### 2.4. Statistical Analysis

For the comparisons of alleles and genotypes frequencies between patients and controls, we used OpenEpi online software, version 3.01 (Two by Two Table statistics) [27]. *p*-values (2-tail from Mid-P exact test) were considered significant if <0.05. The tests for Hardy–Weinberg equilibrium and clinical features association were performed with Plink v 1.07 software [28].

Because this was a hypothesis-driven candidate gene study restricted to two predefined *TNF-alpha* promoter SNPs, we did not apply formal multiple-testing correction. All the other phenotype-stratified analyses were considered exploratory.

### 2.5. Ethics Approval

All patients included in the present study were of Romanian origin. The present study was presented to, evaluated, and approved by the Elias Emergency University Hospital Ethics Committee (registration number 6598; 11 May 2015; Bucharest, Romania) and by the Fundeni Clinical Institute Ethics Committee (registration number 8007; 23 February 2018; Bucharest, Romania). All patients included in the present study signed a written informed consent form before sampling. The study protocol conformed to the ethical guidelines of the 1975 Declaration of Helsinki and its later amendments.

## 3. Results

A total of 358 subjects were included in this study: 198 patients with IBDs and 160 healthy controls. There was no significant difference in the sex ratio between patients with IBDs and controls (*p* = 0.1, OR 0.70, 95% CI 0.45–1.07). The genotyping success rate was 100% for both controls and patients. The distribution of genotypes respected the Hardy–Weinberg equilibrium for controls and patients for the two analyzed SNPs.

The two groups of patients were similar in terms of number of patients studied, sex, and age (Table 1). We noticed a numerically higher usage of anti-TNF agents in the CD group.

The allelic frequencies and the genotypic distribution for the tested polymorphisms in the IBD group, including the UC and CD subgroups, and healthy controls are shown in Table 2A and Table 2B, respectively.

We found a significantly higher frequency of the minor allele A of rs361525 (–238G/A) in patients with CD versus controls (*p* = 0.01, OR = 3.16). The carriers of this allele (AA and GA genotypes) had an increased risk of developing CD (*p* = 0.02, OR = 3.05) in the studied population. For UC and the whole group of patients with IBDs, the frequency of the A allele was also higher than in the controls, but not statistically significantly higher (Table 2B).

When analyzing data regarding the presence of EIMs associated with IBDs, we found that in patients with EIMs, the minor alleles of both SNPs were present at a higher frequency than in the group of patients without EIMs, but this difference was significant only for rs1800629 (for UC, CD, and the whole IBD group, Table 3). For this polymorphism, the genotype frequency analysis showed a significant risk for the carriers of the minor allele of developing EIMs associated with either UC or CD. Half of the patients with EIMs had at least one copy of the rs1800629A allele compared with approximately 10% of the patients without EIMs (*p* = 1 × 10^−4^, OR 9.58; *p* = 9 × 10^−4^, OR 6.60, respectively).

In the whole group of patients with IBDs, the carriers of the minor allele for both SNPs studied (AA+GA) were significantly more likely to have EIMs associated with IBDs (*p* = 3 × 10^−7^, OR 7.87, and *p* = 0.03, OR 3.02, respectively, Table 3B).

Regarding the response to anti-TNF treatment, we found that in the group of primary non-responders, the minor allele for rs1800629 was less frequent than in the group of responders (4.6% versus 10.3%), but the difference was not statistically significant (*p* = 0.1, OR 2.34, 95% CI 0.66–8.25).

The analysis of patients with UC according to the extent of the disease revealed that the frequency of the minor allele of rs1800629 was significantly higher in the subgroup with the E2 phenotype than in those with the E1 and E3 phenotypes (16.6% versus 5.6%, *p* = 0.02, OR 3.32, 95% CI 1.16–9.48).

No associations were found with the other phenotypic characteristics of the two diseases (localization or disease behavior, age at diagnosis, or adverse effects of anti-TNF treatment).

## 4. Discussion

In this study, we investigated the association of two *TNF-alpha* promoter polymorphisms, rs1800629 (–308G>A) and rs361525 (–238G>A), with susceptibility to IBDs and clinical characteristics for the first time in a Romanian cohort. We found that the rs361525 A allele was significantly associated with increased susceptibility to Crohn’s disease (CD), while no such association was observed for ulcerative colitis (UC). This finding is noteworthy given that most prior studies have focused on the rs1800629 variant, often reporting inconsistent results. Some meta-analyses and population studies have reported that the minor −308A allele (rs1800629) is associated with a higher risk of IBDs in certain populations [15,21]. For example, a large meta-analysis by Fan et al. noted that the rs1800629AA genotype significantly increased the risk of both UC and CD in Europeans (OR ≈ 1.7–2.0) [15]. Similarly, a case–control study in Saudi Arabia found the rs1800629A allele to be more frequent in patients with IBDs than in controls [21]. On the contrary, in our study, we observed that the minor allele of rs1800629 was less frequent in patients than in controls; the difference did not reach statistical significance. Other studies have failed to replicate these associations. Mao et al. reported no significant correlation between the rs1800629A allele and CD susceptibility in a meta-analysis encompassing 11 studies (involving Caucasian and Asian populations), and Zipperlen et al. found no association of the either rs1800629A or rs361525A alleles with CD in a Canadian cohort [18,29]. The significant association we observed with the rs361525 A allele in CD, a variant less frequently analyzed in the literature, suggests that *TNF-alpha* promoter polymorphisms beyond rs1800629 contribute to IBD risk in certain genetic backgrounds. This may reflect population-specific genetic heterogeneity that partly explains why our Romanian cohort showed an association of IBD risk with rs361525, whereas other populations have not consistently shown this effect [15,18].

Notably, we found that both *TNF-alpha* promoter SNPs were significantly associated with the development of extraintestinal manifestations (EIMs), analyzed here as a composite phenotype reflecting systemic involvement in IBDs. To the best of our knowledge, this is one of the first reports linking these specific *TNF-alpha* gene variants to EIM susceptibility. EIMs (such as arthritis, uveitis, or skin lesions) are immune-mediated complications of IBDs that substantially increase patient morbidity. Their pathogenesis is associated with systemic inflammation and immune dysregulation extending beyond the gut [30]. TNF-alpha is a key cytokine not only in intestinal inflammation but also in systemic inflammatory pathways, and it has been implicated as a potential driver of EIMs [30]. Our findings, therefore, align with the idea that genetic upregulation of TNF-alpha could predispose patients to extraintestinal immune complications. Although the direct literature on *TNF-alpha* promoter polymorphisms and EIMs is scarce, our results contribute to the understanding that TNF-alpha is an important regulator of systemic inflammation in IBDs [21,22]. Supporting the aforementioned results, a recent large-scale analysis of patients with IBDs identified genetic associations of EIMs in pathways involving TNF-alpha signaling, among others [30]. Thus, it is expected that carriers of *TNF-alpha* genotypes associated with cytokine overexpression (such as rs1800629 GA/AA or rs361525GA/AA) will experience a more intense systemic inflammatory response during disease flares, contributing to an increased likelihood of EIMs.

Our analysis did not reveal any significant association between *TNF-alpha* promoter variants and other clinical parameters such as age at diagnosis, disease location in CD, disease behavior in CD, response to anti-TNF therapy, or history of colectomy. Within the UC group, we observed a signal suggesting a higher frequency of the rs1800629 minor allele in patients with left-sided colitis (E2) than in those with E1 and E3. However, the clinical significance of this extent-restricted finding remains uncertain and should be interpreted cautiously given the limited subgroup sizes and the exploratory nature of phenotype analyses. The lack of genotype–phenotype correlations in our cohort is consistent with that in several previous studies [18,29]. For example, Zipperlen et al. found no relationship between five *TNF-alpha* promoter SNPs and CD disease location or behavior in a Canadian population [18]. Likewise, a meta-analysis did not support a correlation between rs1800629A allele and clinical subtypes of CD overall [29]. However, it is important to acknowledge that some reports have identified associations in specific contexts, contrasting our negative results [31,32]. Sýkora et al. observed in a pediatric cohort with IBDs that the rs1800629A variant was linked to a more severe CD phenotype—specifically, a higher frequency of stricturing/penetrating complications and elevated inflammatory markers in rs1800629 A carriers [31]. In adult patients, Santana et al. similarly reported that the rs1800629A (TNF2) allele, which is associated with higher TNF-alpha production, was more common in patients with CD with penetrating behavior and was associated with an increased likelihood of surgical interventions (e.g., small bowel resection or colectomy) [32].

Given the pragmatic, binary definition of treatment response used in this study (responder vs. primary non-responder), analyses of anti-TNF response were intended as exploratory and should be interpreted accordingly. We did not find an effect of these polymorphisms on response to anti-TNF biologic therapy in our patients, whereas prior pharmacogenetic studies have suggested such an association [14,33]. In our cohort, neither rs1800629 nor rs361525 genotypes were significantly related to treatment response or the need for colectomy, which might reflect limited sample sizes or clinical heterogeneity. Interestingly, a recent study reported that patients with IBDs carrying the rs1800629A allele had a higher likelihood of discontinuing anti-TNF therapy (due to primary non-response or adverse events) than those with the GG genotype [33]. In the same study, the rs361525AA genotype was associated with a lower probability of achieving steroid-free remission on infliximab or adalimumab [33]. Moreover, an earlier analysis in a Spanish cohort noted an increased frequency of the rs1800629A allele among non-responders to infliximab [14]. These findings imply that *TNF-alpha* gene variants influence biologic therapy effectiveness, possibly through changes in baseline TNF-alpha levels or other immune mechanisms that determine the dependency of a given patient’s disease on TNF-alpha signaling. Our negative results on therapy response, therefore, should be interpreted with caution. It may be that our study was underpowered to detect what are likely small-to-moderate effect sizes.

Our findings should be interpreted with caution considering this study’s many limitations. The sample size, especially for subgroup analyses (patients with EIMs and patients assessed for therapy response), was moderate, which may have limited the detection of weaker associations and contributed to false-negative results. As stated above, the continuous consecutive inclusion strategy precluded us from adhering to a formal precalculated power analysis. The study population was confined to Romanian patients, and, while this homogeneity minimized population stratification, it may affect the generalization of the results. Allele frequency differences across ethnicities are well-documented for *TNF-alpha* polymorphisms [15]. For instance, the rs1800629A allele is relatively uncommon in East Asian populations but more prevalent in European groups [15]. Thus, replication across diverse cohorts is necessary to determine whether the associations observed (such as that of rs361525A with CD or the polymorphisms with EIMs) persist elsewhere. Another limitation is that we focused on two promoter SNPs and did not examine other *TNF-alpha* gene variants (e.g., –857C>T or –1031T>C) that have been studied by other researchers [18]. Another possibility is that the effect of rs1800629A or rs361525A may be modified by or linked to other polymorphisms in the TNF locus or adjacent genes in the HLA region. Additionally, we did not measure circulating or mucosal TNF-alpha levels in our patients. Integrating functional assays such as cytokine measurements is an important next step to bridge the gap between genotype and phenotype. For example, demonstrating that patients with the risk alleles indeed have higher TNF-alpha expression or a more florid inflammatory response would strengthen the causal inference between these polymorphisms and disease manifestations. Lastly, environmental and clinical factors (such as smoking status, microbiome composition, or treatments received) were not included in our analysis. All these factors could confound or interact with genetic effects and deserve consideration in future studies. Moreover, extraintestinal manifestations may correlate with disease severity and treatment exposure; because we relied on univariate comparisons, we could not formally adjust for these potential confounders, and the EIM-related associations should therefore be interpreted as exploratory. Because the *TNF* promoter variants analyzed are located within the extended MHC region, the observed associations may partially reflect linkage disequilibrium with nearby HLA haplotypes rather than direct functional effects of the *TNF* polymorphisms. In addition, the absence of ancestry-informative markers or HLA typing precluded a formal assessment of population stratification and linkage disequilibrium effects within the TNF/HLA region, particularly for low-frequency variants such as rs361525.

In summary, our study contributes to the existing evidence that host genetic variability in the *TNF-alpha* gene can influence IBDs. We report that the *TNF-alpha* rs361525 promoter polymorphism was associated with CD susceptibility and that both rs1800629 and rs361525 variants were linked to EIMs in a Romanian cohort. These results highlight TNF-alpha’s relevance not only in intestinal inflammation but also in the systemic features of IBDs. At the same time, the lack of association with most of the clinical phenotypes and treatment response points towards the complexity of IBD genetics. Thus, the contribution of any single cytokine gene variant is likely modest and may be obscured by the polygenic nature of the disease and environmental modifiers. From a clinical perspective, our findings alone are not sufficient to alter management. Nevertheless, as research advances, a better understanding of *TNF-alpha* polymorphisms could aid in risk stratification or identifying subsets of patients who might benefit from personalized therapy. Ultimately, our results contribute to the growing literature on IBD pathogenesis and support the ongoing exploration of TNF-alpha-targeted strategies. Further large-scale, multi-center studies, incorporating genomic, transcriptomic, and clinical data, are necessary to validate these associations and to elucidate how *TNF-alpha* gene variations interact with other factors to shape the IBD phenotype.

## 5. Conclusions

Our study shows that the *TNF-alpha* promoter polymorphism rs361525 is associated with susceptibility to develop Crohn’s disease, while both rs1800629 and rs361525 are linked to the occurrence of extraintestinal manifestations in IBD patients from Romania. These findings highlight the role of *TNF-alpha* genetic variants in disease susceptibility and systemic involvement, supporting their potential relevance in understanding clinical phenotype heterogeneity within IBDs.

## Figures and Tables

**Table 1:** Demographic data of the IBD group.

	Crohn’s Disease (CD)	Ulcerative Colitis (UC)
Patients N (%)	106 (53.5%)	92 (46.5%)
Sex (M/F)	57/49	55/37
Age at disease onset (mean, years)	29.7	34.4
Disease extension (Montreal Classification)	L1: 46	E1: 9
	L2: 26	E2: 48
	L3: 34	E3: 35
Disease behavior (for CD)	B1: 49	N/A
	B2: 36	N/A
	B3: 21	N/A
Anti-TNF treatment (Y/N)	83/23	37/55
Response to anti-TNF treatment (Y/N)	64/19	24/13
Adverse effects to anti-TNF treatment N (%)	12 (14.5%)	1 (2.7%)
Presence of extraintestinal manifestations N (%)	24 (22.6%)	21/89 (23.6%) *

* Data available for 89 patients with UC. Y/N—yes/no.

**Table 2:** (**A**). Results of the allele-based association study of *TNF-alpha* single-nucleotide polymorphisms (SNPs) and IBDs. (**B**). Results of the genotype-based association study of *TNF-alpha* single-nucleotide polymorphisms (SNPs) and IBDs.

(A)								
SNP (*TNF-alpha*)	Controls: Minor Allele A, *n* (%)		UC: Minor Allele A, *n* (%)	UC vs. Controls, OR (95% CI), *p*	CD: Minor Allele A, *n* (%)	CD vs. Controls, OR (95% CI), *p*	IBDs: Minor Allele A, *n* (%)	IBDs vs. Controls OR (95% CI), *p*
rs1800629	42 (13.1%)		21 (11.4%)	0.85 (0.48–1.49), *p* = 0.5	19 (8.9%)	0.65 (0.36–1.15), *p* = 0.1	40 (10.1%)	0.74 (0.46–1.17), *p* = 0.2
rs361525	7 (2.2%)		5 (2.7%)	1.24 (0.39–3.99), *p* = 0.7	14 (6.6%)	3.16 (1.25–7.96), ***p* = 0.01**	19 (4.8%)	2.25 (0.93–5.43), *p* = 0.06
**(B)**								
**SNP** ***(TNF-alpha)***	**Genotype**	**Controls,** ***n*** **(%)**	**UC,** ***n*** **(%)**	**UC vs. Controls, OR (95% CI),** ***p***	**CD,** ***n*** **(%)**	**CD vs. Controls, OR (95% CI),** ***p***	**IBDs,** ***n*** **(%)**	**IBDs vs. Controls, OR (95% CI),** ***p***
rs1800629	AA + GA	37 (23.1%)	18 (19.5%)	0.80 (0.42–1.52), *p* = 0.5	18 (17.0%)	0.68 (0.36–1.27), *p* = 0.2	36 (18.1%)	0.73 (0.44–1.23), *p* = 0.2
	GG	123 (76.9%)	74 (80.5%)	Reference	88 (83.0%)	Reference	162 (81.9%)	Reference
rs361525	AA + GA	7 (4.3%)	5 (5.4%)	1.25 (0.38–4.07), *p* = 0.7	13 (12.2%)	3.05 (1.17–7.93), ***p* = 0.02**	18 (9.0%)	2.18 (0.88–5.37), *p* = 0.08
	GG	153 (95.7%)	87 (94.6%)	Reference	93 (87.8%)	Reference	180 (91.0%)	Reference

(A) Values represent minor allele A counts, expressed as n (%). OR indicates association of A vs. G using controls as the reference group. (B) Values represent carriers of the minor allele A (AA+AG) counts, expressed as n (%). OR indicates association for A-allele carriers (AA/GA) versus GG (dominant model), using controls as the reference group. *p* values < 0.05 are indicated in bold; OR, odds ratio; 95% CI, 95% confidence interval.

**Table 3:** (**A**). Results of the allele-based association study of *TNF-alpha* single-nucleotide polymorphisms (SNPs) and the presence of EIMs. (**B**). Results of the genotype-based association study of *TNF-alpha* single-nucleotide polymorphisms (SNPs) and the presence of EIMs.

(A)										
SNP (*TNF-alpha*)		UC Without EIMs: Minor Allele A, *n* (%)	UC with EIMs: Minor Allele A, *n* (%)	OR (95% CI), *p*	CD Without EIMs: Minor Allele A, *n* (%)	CD with EIMs: Minor Allele A, *n* (%)	OR (95% CI), *p*	IBDs Without EIMs: Minor Allele A, *n* (%)	IBDs with EIMs: Minor Allele A, *n* (%)	OR (95% CI), *p*
rs1800629		10 (7.3%)	11 (26.2%)	4.47 (1.74–11.47), ***p* = 0.002**	8 (4.8%)	11 (22.9%)	5.79 (2.17–15.43), ***p* = 5 × 10^−4^**	18 (6.0%)	22 (24.4%)	5.06 (2.57–9.97), ***p* = 4 × 10^−6^**
rs361525		2 (1.4%)	3 (7.1%)	5.15 (0.83–31.95), *p* = 0.09	9 (5.4%)	5 (10.4%)	2.00 (0.63–6.28), *p* = 0.2	11 (3.6%)	8 (8.8%)	2.56 (0.99–6.58), *p* = 0.06
**(B)**										
**SNP ** ** (*TNF-α*)**	**Genotype**	**UC Without EIMs,** ***n*** **(%)**	**UC with EIMs,** ***n*** **(%)**	**OR (95% CI),** ***p***	**CD Without EIMs,** ***n*** **(%)**	**CD with EIMs,** ***n*** **(%)**	**OR (95% CI),** ***p***	**IBDs Without EIMs,** ***n*** **(%)**	**IBDs with EIMs,** ***n*** **(%)**	**OR (95% CI),** ***p***
rs1800629	AA + GA	7 (10.3%)	11 (52.4%)	9.58 (3.00–30.57), ***p* = 1 × 10^−4^**	8 (9.7%)	10 (41.6%)	6.60 (2.21–19.67), ***p* = 9 × 10^−^**^4^	15 (10.0%)	21 (46.6%)	7.87 (3.56–17.39), ***p* = 3 × 10^−^**^7^
	GG	61 (89.7%)	10 (47.6%)	Reference	74 (90.2%)	14 (58.3%)	Reference	135 (90.0%)	24 (53.3%)	Reference
rs361525	AA + GA	2 (3.0%)	3 (14.3%)	5.50 (0.85–35.45), *p* = 0.09	8 (9.7%)	5 (20.8%)	2.43 (0.71–8.29), *p* = 0.1	10 (6.6%)	8 (17.7%)	3.02 (1.11–8.21), ***p* = 0.03**
	GG	66 (97.0%)	18 (85.7%)	Reference	74 (90.2%)	19 (79.2%)	Reference	140 (93.4%)	37 (82.2%)	Reference

(A) Values represent minor allele A counts expressed as n (%). OR indicates association of EIMs present vs. absent using an allelic model (A vs. G) within each disease group. (B) Values represent carriers of the minor allele A (AA+AG) counts, expressed as n (%).OR indicates association for A-allele carriers (AA/GA) versus GG (dominant model), comparing EIMs present vs. absent within each disease group. UC—ulcerative colitis; CD—Crohn’s disease; IBDs—inflammatory bowel diseases, EIMs—extraintestinal manifestations. OR—odds ratio; CI, 95%—confidence interval; *p* values < 0.05 are indicated in bold.

## Data Availability

The raw data supporting the results are available from the corresponding author upon reasonable request.
